# Targeting Trimethylamine N-Oxide: A New Therapeutic Strategy for Alleviating Atherosclerosis

**DOI:** 10.3389/fcvm.2022.864600

**Published:** 2022-06-13

**Authors:** Lele Jing, Honghong Zhang, Qiannan Xiang, Liang Shen, Xiaoxia Guo, Changlin Zhai, Huilin Hu

**Affiliations:** ^1^Department of Cardiology, The Affiliated Hospital of Jiaxing University, Jiaxing, China; ^2^School of Clinical Medicine, Zhejiang Chinese Medical University, Hangzhou, China

**Keywords:** atherosclerosis, gut flora, flavin-containing monooxygenase-3, trimethylamine, trimethylamine N-oxide

## Abstract

Atherosclerosis (AS) is one of the most common cardiovascular diseases (CVDs), and there is currently no effective drug to reverse its pathogenesis. Trimethylamine N-oxide (TMAO) is a metabolite of the gut flora with the potential to act as a new risk factor for CVD. Many studies have shown that TMAO is involved in the occurrence and development of atherosclerotic diseases through various mechanisms; however, the targeted therapy for TMAO remains controversial. This article summarizes the vital progress made in relation to evaluations on TMAO and AS in recent years and highlights novel probable approaches for the prevention and treatment of AS.

## Introduction

Cardiovascular disease (CVD) is presently the most harmful type of disease in the world. Traditional risk factors for CVD include hypertension, hyperlipidemia, diabetes, obesity, smoking, etc. (1). Research has shown that strengthening control over risk factors and reducing exposure to risk factors can significantly reduce the incidence of CVD, as well as mortality from it (2–4). However, even with tight control over traditional risk factors, a considerable proportion of patients remain vulnerable to high risks of cardiovascular events. Therefore, there is an urgent need for the identification of new pathogenic factors.

Trimethylamine N-oxide (TMAO) is a metabolite of the intestinal flora that appears to play a major role in CVD. A large cohort study of 4,007 cases found differences in plasma TMAO levels between CVD patients (5.0 μM) and healthy individuals (3.5 μM). Under the premise of the same traditional risk factors for CVD, patients with high plasma TMAO levels (more than 6.18 μM) had higher incidences of major adverse cardiovascular events in 3 years than patients with low plasma TMAO levels (5), pointing to TMAO as a possible new risk factor for CVD.

Atherosclerosis (AS) is one of the most common CVDs, bringing huge social and economic burdens on countries. Studies have shown that AS is closely linked to pathological changes, such as lipid metabolism disorders, inflammatory responses, and oxidative stress (6, 7). Recent investigations have confirmed that intestinal flora disorders can cause metabolic ailments and inflammatory reactions through metabolism and the immune system, leading to the formation and rupture of atherosclerotic plaques (8, 9). Other inquiries have demonstrated that lipopolysaccharides (LPS) from E. coli can enhance platelet aggregation via Toll-like receptor (TLR)4-mediated leucocyte cathepsin G activation, and multiple effects of LPS may converge to accelerate atherogenesis (10, 11). Microbiota can also influence platelet function through hepatic TLR2 signaling (12). A gut microbiota-derived metabolite, phenylacetylglutamine (PAGln), reportedly enhances platelet activation-related phenotypes and thrombosis potential (13). In addition, associated studies have noted that the gut microbiota also promotes arterial thrombosis through the following mechanisms: adhesion-induced platelet activation (14), direct or indirect enhancement of human platelet responsiveness to multiple agonists (15, 16), enhancement of platelet deposition to subendothelial matrix molecules (17).

Microbiota influence the development of AS in a dietary-dependent manner. Some animal model studies have established no difference in atherosclerotic lesion size between specific pathogen-free (conventionally raised, CONV-R) and germ-free (GF) mice on a high-fat diet (HFD) (14, 18), while others have noted bigger atherosclerotic lesion sizes in GF mice than in CONV-R mice on chow (18, 19). These outcomes point to a protective effect of microbiota (commensal bacteria) on AS development, a role also reported by the Kappel BA group, who found an association between increased AS caused by antibiotics and the loss of intestinal diversity. The commensal microbiota comprises both pathogenic and protective bacteria, and not all gut microbiota metabolites are pathogenic.

Short-chain fatty acids (SCFAs) are a class of saturated fatty acids produced by anaerobic bacteria or yeast. Reports on the beneficial roles of SCFAs in AS are becoming increasingly numerous. Some data suggest that SCFAs have atheroprotective effects, among which butyrate exerts AS protection through multiple mechanisms (20–23).

Even though a connection has been established between circulating TMAO and the risk of cardiovascular events, the role of TMAO in AS remains controversial, with the mechanism not yet fully elucidated. Here, we discuss the relationship and related pathophysiologic mechanisms between TMAO and AS, as well as the potential therapeutic strategies that target these pathophysiologic mechanisms. This review aims to provide a new perspective for finding new approaches to prevent and treat AS.

## The Source, Synthesis, and Metabolism of Tmao

### The Source and Synthesis of TMAO in the Human Body

TMAO is widely found in aquatic products in nature, as well as in other animals, plants, and even fungi. In the human body, TMAO is an intestinal microbiota-dependent metabolite from dietary phosphatidylcholine (5), L-carnitine (24), and betaine (25).

Typically, nutrients, such as choline, are absorbed through receptors in the small intestine. When the availability of nutrients to be taken up exceeds the absorption capacity of the small intestine, excess nutrients end up in the large intestine. Intestinal microbes in the large intestine have choline trimethylamine (TMA)-lyase (CutC), which can break the CN bonds in nutrients to produce the tasteful TMA gas (26). CutC is the key enzyme for TMA production. Colonizing the mouse gut with a specific consortium of CutC-encoding human isolates results in TMA synthesis and subsequent TMAO accumulation in the serum of animals, where even minute concentrations of TMA producers are sufficient for substantial TMA production from choline (27). The produced TMA quickly enters the circulatory system through the blood vessel wall (28, 29). When TMA reaches the liver via portal circulation, it is oxidized to odorless TMAO by flavin-containing monooxygenase-3 (FMO3) and then re-enters blood circulation, ending up in body tissues.

Plasma TMAO levels in healthy humans are about 0.5–5.0 μM (30), and in patients with renal failure, TMAO levels can reach to 40 μM (31), possibly due to the high intake of TMAO precursor substances and the low excretion rate of the kidneys. Additionally, age factors, cholic acid levels, and sex hormone levels can affect the level of TMAO (32). Cholic acid and sex hormones can further alter the quantity of TMAO in the body by upsetting the activity of FMO3 (33).

### The Metabolism of TMAO

TMAO is a small molecule compound and is, therefore, easily filtered by the kidneys (34). A study on the populations of different countries found that Swiss people who ate more fish had higher levels of TMAO in urine than their British counterparts. Consistent with this result, Japanese people had higher levels of TMAO in urine than North Americans. Hence, a high-protein diet also increases the level of TMAO in urine (31), perhaps because the high-protein diet changes the structure of the intestinal flora or upsets the absorption of choline and L-carnitine in the intestine. In addition to being excreted in the urine, TMAO can be eliminated through sweat, feces, and the respiratory tract (33). In most cases, after 24 h of metabolism in sweat, urine, and breathing, 50% of TMAO cannot be removed, with the excess TMAO reconverted into TMA under the action of the intestinal flora TMAO reductase (35).

FMO3 is the key enzyme for the transformation of TMA to TMAO in the liver, and its transformation efficiency is the highest. Mutations in *FMO3* can prevent TMA from being converted into TMAO, with excessive TMA excreted through urine, sweat, and breathing, emitting a fishy smell, which is clinically called fishy smell syndrome (36).

### TMAO's Impact on AS

In recent years, TMAO has been found to be associated with heart failure (37), chronic kidney disease (38, 39), tumors, obesity (40), diabetes (41), and other diseases. Studies have shown that TMAO (21 μM in male mice and more than 70 μM in female mice) enhances the development of AS lesions in mice and that plasma TMAO levels correlate significantly positively with the size of AS plaques (29). Altering intestinal flora and inhibiting other pathways to reduce plasma TMAO levels can drastically repress the inflammation of vascular endothelial cells and the formation of macrophage-derived foam cells, as well as reduce the area of mouse aortic AS (9, 24, 29).

Controversially, a study on Apoe^−/−^ mice revealed that choline supplementation promotes increased TMAO levels (increase to 20 μM on chow diet and 9 μM on Western diet) in CONV-R mice but does not affect aortic lesion size, whether on chow or HFD (18). Kiouptsi K et al. also reported no change in the absolute size of late atherosclerotic plaques in the carotid artery of HFD-fed GF Ldlr^−/−^ mice and HFD-fed CONV-R Ldlr^−/−^ mice (14). Paulina et al. recorded no alteration in atherosclerotic lesion size related to high intakes of dietary choline or TMAO supplementation, irrespective of the mouse model (42). However, these experimental settings notably differ from previous ones, given that they supplemented choline or TMAO at a later time; therefore, the impact of choline or TMAO on early AS development cannot be ruled out.

Some clinical studies have also suggested that TMAO has a positive role in atherosclerotic disease. A BioHEART-CT study uncovered a negative association of plasma TMAO with the presence of soft plaque, total plaque burden, and calcified plaque burden in univariate and multivariable models after adjusting for age, sex, and traditional risk factors (43). A CARDIA investigation indicated that TMAO may not contribute significantly to advancing early atherosclerotic disease risk in healthy early-middle-aged adults (44). Bordoni et al. recently found the link between high TMAO and CAD to be peculiar to the rs247616-CC risk genotype (cholesterol ester transfer protein) but not generalizable to the entire population (45). Both genes and the environment participate in the process of TMAO's influence on AS, so these effects are markedly individualized.

Despite the controversy, this manuscript intends to clarify the mechanism of action of TMAO in AS and attempt to suit the degree of doubt.

### TMAO Activates Inflammation

AS is a chronic inflammatory disease that involves a variety of inflammatory factors. Seldin et al. observed that the aortas of Ldlr^−/−^ mice fed a choline diet showed elevated inflammatory gene expression compared to controls (46). To confirm the role of TMAO in cellular inflammation, they injected 86 μmol of TMAO into Ldlr^−/−^ mice, and half an hour later, the activation of p38 mitogen-activated protein kinase (p38 MAPK), extracellular signal-regulated kinase 1/2 (ERK1/2), and nuclear factor kappa-B p65 (NF-κB p65) cascade signaling were detected in aortic samples (46). *In vitro*, they demonstrated TMAO's induction of the expression of inflammatory genes, including cyclooxygenase 2, interleukin 6, etc., both in human endothelial and smooth muscle cells, which eventually caused vascular endothelial inflammatory damage and enhanced the adhesion of leukocytes to vascular endothelia (46). Chen et al. found that TMAO promoted vascular inflammation by activating the nucleotide-binding oligomerization domain-like receptor family pyrin domain-containing 3 (NLRP3) inflammasome in human umbilical vein endothelial cells and aortas from Apoe^−/−^ mice (47).

### TMAO Increases the Risk of Thrombosis

Zhu et al. found that TMAO enhanced thrombosis risk by reanalyzing the clinical data of 4,007 subjects (15). Using a mouse carotid artery injury (FeCl3) model, they found that exposure to a physiological dose of TMAO (100 μmol) accelerated the formation of arterial thrombosis and shortened the duration of vascular occlusion (15), possibly due to the occurrence of acute coronary syndrome. Examinations of the underlying mechanism of this phenomenon suggested that this physiological dose of TMAO directly enhanced platelet reactivity to adenosine diphosphate, thrombin, and collagen and amplified platelet adhesion to collagen (15). Cellular experiments have shown that rapid (10 min) exposure to a physiological dose of TMAO enhances platelet endogenous calcium release dose-dependently (15). Based on these prothrombotic mechanisms, some researchers have attributed the resistance of clopidogrel to TMAO (48).

Recent investigations have demonstrated that TMAO enhances platelet reactivity by promoting ERK1/2 and Jun N-terminal kinase (JNK) phosphorylation (49). *In vivo* and *in vitro* experiments have revealed that TMAO increases thrombotic potential by strengthening aortic vascular tissue factor (TF) and vascular cell adhesion molecule (VCAM)1 expression (50).

### TMAO Promotes Foam Cell Formation

Foam cells are the main component of atherosclerotic plaques. Monocytes adhering to the surface of endothelial cells migrate into the media and transform into macrophages, which swallow large amounts of oxidized low-density lipoprotein cholesterol through scavenger receptors on the cell surface to form foam cells.

Ma and colleagues reported that TMAO stimulates monocyte adhesion to human umbilical vein endothelial cells (HUVECs) in a dose-dependent manner early during foam cell formation. Adhesion assay findings on the aorta of C57BL/6 mice reinforced the deduction that TMAO promotes the adhesion of monocytes to the aorta, with a maximum dose of TMAO at 10 mmol/L (51). TMAO can also up-regulate the number of scavenger receptors on the surface of macrophages (52). Three weeks of 0.12% TMAO supplementation during weaning in C57BL/6 and Apoe^−/−^ mice significantly increased the expression of scavenger receptors CD36 and SR-A1 in peritoneal macrophages compared to a normal chow diet (29). In addition, elevated serum TMAO levels alter static electricity at the endothelial cell membrane-blood interface (arterial wall), potentially affecting the inflow/efflux of fatty deposits on the arterial wall (53). Under the combined action of these mechanisms, foam cell formation proliferates, providing a material basis for the development of atherosclerotic plaques.

### TMAO Reduces Cholesterol Metabolism

The impact of cholesterol concentration on AS is remarkably distinct. The main cholesterol metabolic pathway in the body is reverse transport to the liver, where bile acids are synthesized. Animal experiments have shown that choline or carnitine supplementation markedly (approximately 30%) reduces reverse cholesterol transport (RCT) in mice compared to normal chow controls, a process that can be completely inhibited by oral broad-spectrum antibiotics (24). Some investigations have also established differences in cholesterol levels between TMAO mice and control mice, with one inquiry showing that the expression of cholesterol 7α-hydroxylase (Cyp7a1) in TMAO-intervention mice was 38.4% lower than that in control diet mice, indicating a specific decline in the classical bile acid synthesis pathway (54). The collective impact of TMAO on RCT and cholesterol-bile acid metabolic pathways leads to an increase in blood cholesterol, promoting foam cell formation and aggravating AS.

## Preventions and Treatments Targeting TMAO

Because TMAO is possibly a new high-risk factor for AS diseases, the prevention and treatment of AS through targeting TMAO require increased attention, which this paper provides as follows.

### Dietary Intervention

One World Health Organization report (55) advises that 60% of human health depends on personal lifestyle, and only 40% depends on genetic inheritance, medical conditions, social environment, and other factors. Therefore, the most straightforward intervention is to adjust diets. Limiting excessive intake of foods rich in phosphatidylcholine, choline, and carnitine could be an effective strategy to limit circulating TMAO levels.

Tests show that TMAO levels surge with increasing choline intake and age (32); fasting plasma TMAO levels and urine TMAO levels of vegetarians are lower than those of omnivores. 16s rRNA sequencing of fecal samples revealed higher abundances of Clostridia and Peptostreptococcaceae in omnivores, but lower abundances of Trichospira and Bacillus (24). Reducing the number of L-carnitine and phosphatidylcholine-containing products in the diet of patients with coronary artery disease could positive the decrease in the proatherogenic metabolite TMAO concentration (56). Therefore, dietary intervention possibly upsets the production of TMAO by changing the composition ratio of the intestinal flora.

One investigation on dietary interventions (57) in healthy subjects divided into a VEG group (usual dietary regimen + 4 servings of vegetables) and an FMD group (5-day hypocaloric diet) established that the plasma TMAO, fasting blood glucose, and C-peptide of subjects in the FMD group diminished considerably compared to the VEG group. This finding suggests intermittent fasting as a viable option for improving cardiometabolic health. Studies have also shown that a vegan diet is an effective strategy for improving glucose tolerance and reducing plasma TMAO in individuals with dysglycemia or obesity (58). A ketone diet (KD) can alter the structure and function of the intestinal microbial group (59); however, there is no published literature exploring the relationship between KD and TMAO in AS.

Numerous inquiries have pointed to the Mediterranean diet (MD) as a strategy for preventing and reducing the risk of cardiovascular and metabolic diseases (60–62). The recent CORDIOPREV study found MD to decrease the progression of AS in patients with coronary heart disease (63). Although the MD is rich in TMAO, there is every indication that the MD does not affect plasma TMAO concentrations in healthy people (64) or lower plasma TMAO concentrations (65–67), perhaps because the MD modulates the composition of gut microbiota (66). Dietary apigenin reverses multiple pro-atherosclerotic mechanisms involved in TMAO, including inflammasomes, low-density lipoprotein uptake, leukocyte adhesion, etc. (68). Reports on animal experiments show that *Ligustrum robustum* (a new food resource) reduces serum TMA and TMAO levels by moderating gut microbiota, increasing fecal cholesterol excretion, decreasing serum and liver cholesterol levels, and ultimately attenuating diet induced AS (69).

So far, the need to fundamentally limit the intake of TMAO precursors to alter the production of TMAO remains unclear. Nonetheless, recent studies have found that nutrients containing TMA are essential for survival and have a protective effect on the heart. Choline and betaine can prevent CVD by reducing inflammation. L-carnitine diminishes the incidence of angina pectoris by 40% and all-cause mortality caused by acute myocardial infarction by 27% (70). Dietary phosphatidylcholine supplementation lessens AS in Ldlr^−/−^ male mice (71). The balance between the cardiovascular benefits of nutrients containing TMA and the risk of TMAO formation should be the focus of future research.

### Regulating the Intestinal Flora

The intestinal flora is composed of a variety of bacteria and participates in the metabolism of the human body. Time-dependent increases in the levels of both TMAO and its d9 isotopolog, as well as other choline metabolites, are detectable after a phosphatidylcholine intervention. Plasma TMAO levels are markedly suppressed after the use of antibiotics but reappear after the withdrawal of antibiotics (5). Antibiotics and other drugs can reverse the formation of arterial plaques (29). Therefore, using antibiotics to change the composition of the intestinal flora or metabolic activity to reduce plasma TMAO levels is a probable method for preventing and treating AS. However, the adverse consequences of the long-term use of antibiotics could become an obstacle to this targeted therapy. Long-term use of antibiotics can cause an imbalance in the intestinal flora and fungal infections, as well as lead to the emergence of drug-resistant bacteria and insulin resistance.

Probiotics colonized in the intestine can improve balance in a host's micro-ecology and delay the progression of AS (72). Lactobacillus can regulate the structure of the intestinal flora and its metabolites, improving lipid metabolism and plasma TMAO levels (73). Qiu et al. demonstrated that *L. plantarum* ZDY04 is a potential alternative approach to reducing plasma TMAO levels and TMAO-induced AS in mice (73). Probiotic Bif. Animalis subsp. Lactis F1-3-2 intervention can cause a decrease of TMA levels in the cecum of mice and an improvement of lipid metabolism by acting on farnesoid X receptors and cholesterol 7-alpha hydroxylase (74). A recent investigation found B. breve Bb4 and B. longum BL1 and BL7 to significantly diminish plasma TMAO and plasma and cecal TMA concentrations (75).

Boutagy et al. tried decreasing TMAO levels in the intestinal flora using probiotic supplements but noted that adding the multi-strain probiotic VSL#3 failed to alter plasma TMAO levels and did not affect the plasma concentration of L-carnitine, choline, or betaine (76). In another research, a higher proportion of participants in the probiotic group had reduced TMAO after probiotic intervention (77). Different probiotics have different abilities to metabolize choline, which may lead to contradictory research results. Therefore, antibiotics or probiotics targeting choline metabolism-related bacteria should constitute the direction of future research.

### Inhibiting TMAO Precursor Production

CutC is a key enzyme that regulates choline anaerobic metabolism. Research has shown that the microbial colonization of a CutC containing human commensal microbes within a defined (ΔcutC) microbial community transmits into recipients' TMA/TMAO generation, heightened platelet responsiveness, and enhanced thrombosis potential. The microbial choline TMA lyase pathway could be considered a plausible molecular target for the treatment of atherosclerotic heart disease (78). Methods to quantify the potential of human fecal samples to produce TMA have been developed, providing an effective tool for establishing specific therapeutic strategies to inhibit TMA production (79, 80).

3, 3-Dimethyl-1-butanol (DMB) is a structural analog of choline that appears to have certain mitigating properties on TMAO and other effects. After extracting DMB from red wine, Wang et al. confirmed that supplementing a high-cholinergic diet with it can reduce plasma TMAO levels (from 22.1 μM to 15.9 μM), inhibit the formation of foam cells in mice, and prevent the progression of aortic root plaques (81). Another study also reported DMB's ability to attenuate choline diet-enhanced platelet responsiveness and *in vivo* rate of thrombus formation (16). DMB reduces TMA generation through two main mechanisms (81): the direct inhibition of microbial choline TMA lyase activity and the reduction of the proportion of TMA-producing specific bacteria in the intestinal flora. DMB can act on TMA lyase without killing the intestinal flora, by which the physiological functions of the intestinal flora are preserved. There is a considerable application prospect of DMB in inhibiting the generation of TMA/TMAO.

New choline TMA lyase inhibitors, including iodomethylcholine (IMC) and fluoromethylcholine (FMC), have been developed. IMC and FMC suppress host TMA and TMAO levels for sustained periods, with limited systemic exposure and without notable toxicity, at inhibitory efficiencies of about 10,000 times that of DMB (16). Existing reports show that IMC can suppress choline diet-enhanced platelet aggregation and the *in vivo* rate of thrombus formation without increased bleeding time (16), and it can also alter host cholesterol and bile acid metabolism in preclinical animal models (82). In addition, a TF-inhibitory antibody or FMC can abrogate TMAO-dependent enhancements of *in vivo* TF expression and thrombogenicity (50). Reportedly, Meldonium lowers the production of TMA by inhibiting the L-carnitine metabolism intermediate γ-butyl betaine (83).

In summary, targeting the inhibition of TMA lyase could reduce plasma TMAO levels and play a protective role in AS. With these powerful pharmacologic tools in hand, there is now a realistic possibility that TMAO-lowering drugs can be advanced from preclinical models into human studies.

### Inhibiting the Conversion of TMA

FMO3 is the key enzyme in the conversion of TMA to TMAO. Blocking the TMA/FMO3/TMAO pathway is another approach to scaling down the production of TMAO. Shih et al. found that plasma TMAO levels in *FMO3* knockout mice decreased (decreased by about 50% as compared with the control ASO-treated mice), leading to a reduced AS burden (84). Other tests have demonstrated that naringin, paeoniflorin, β-ecdysterone, 18β-glycyrrhizic acid, amygdalin, albiflorin, and saikosaponin A can downregulate FMO3 activity and reduce TMAO biosynthesis (85). Cashman et al. noted that the acid condensation product of indole-3-methanol is an effective inhibitor of FMO3, which can decrease human plasma TMAO levels (86). Recent investigations have shown that trigonelline, a compound extracted from the plant fenugreek, can obstruct FMO3, thereby preventing the conversion of TMA to TMAO (87). *In vitro* evaluations have revealed that 300 μg/mL of trigonelline can trigger its maximum inhibitory effect, reducing the production of TMAO by 85.3% (87).

FMO3, via its participation in TMAO generation, is a regulator of both platelet responsiveness and *in vivo* thrombosis potential (88). Moreover, using an antisense oligonucleotide (ASO) knockdown strategy to target FMO3 results in the reduction of TMAO, platelet responsiveness, and thrombosis potential (89). Inhibiting FMO3 expression in the liver or decreasing FMO3 activity with drugs is another technique for lowering plasma TMAO levels and delaying the progression of AS. However, FMO3 inhibitors that do not cause significant liver damage must be sought.

### Fecal Microbiota Transplantation

Fecal microbiota transplantation (FMT) refers to removing stool from a healthy person and implanting it in a patient's intestine during endoscopy to treat diseases. FMT is currently mainly used to treat inflammatory bowel disease, irritable bowel syndrome, constipation, metabolic syndrome, and other diseases. One randomized placebo-controlled trial that examined metabolic syndrome patients for the effect of bacterial transplantation on the treatment of the disease established changes in the structure of the intestinal flora of the two groups of patients 2 weeks after transplantation but no alterations in the ability to produce TMAO (90). GREGORY et al. found susceptibility to AS to possibly be transmitted through FMT (91). TMA/TMAO production and thrombotic risk phenotypes could equally be transmitted through a human fecal transplant to a germ-free recipient (78).

FMT is potentially an effective measure to reduce the level of circulating TMAO and the risk of AS. However, there are few related studies at present. Because FMT therapy could result in immune rejection in the recipient and ethical issues, researchers need to be cautious with its application.

### Medicine

Various drugs have been shown to modulate the gut microbiota. Feces from donor mice treated with metformin were transplanted into recipient mice, and results showed altered gut microbiota and improved glucose tolerance in the recipient mice (92). More direct evidence indicates that the gut microbiota of Apoe^−/−^ mice are adjusted, short-chain fatty acids are up-regulated, and the degree of AS is mitigated after 12 weeks of metformin intervention (93). However, (92) did not address the impact of metformin on TMAO.

Resveratrol, a non-flavonoid polyphenolic organic compound, is now used as a drug, with studies showing that it attenuates TMAO-induced AS by lowering TMAO levels and increasing hepatic bile acid synthesis via remodeling the gut microbiota (94). Other investigations show resveratrol to have no effect on plasma TMAO levels in mice fed a choline-supplemented diet (16). Notably, drugs targeting TMAO to reverse AS have not yet been developed, and more preclinical and clinical studies are needed.

### Traditional Chinese Medicine

Progressively more studies are revealing that effective ingredients of Chinese medicines, such as Berberine (95), Ganoderma (96), and some Chinese medicine prescriptions (97–99), can adjust the structure of the intestinal flora, reduce inflammation, improve metabolic abnormalities, and delay the occurrence and development of AS. The mechanism through which these ingredients act probably occurs in the following manner: the biologically active ingredients in traditional Chinese medicine alter the structure of the intestinal flora, the endogenous metabolites produced by some traditional Chinese medicines with the participation of the intestinal flora play an anti-inflammatory and anti-AS effect, Chinese medicine adjusts the intestinal flora imbalance caused by AS to improve AS.

Shi et al. noted that Apoe^−/−^ mice exhibited changes in their intestinal flora, significant decreases in the expression of hepatic FMO3 and the concentration of TMAO in plasma, and attenuated HFD-induced AS (100). The underlying mechanism of these alterations possibly lies in berberine inhibiting the anaerobic synthesis of TMA in the intestinal flora (101). Alisma orientalis beverage, a Chinese traditional medicine formulated with a diversity of medicinal plants, reportedly has similar effects to berberine (102). Ganoderma can increase the abundance of Firmicutes and Proteobacteria, decrease the plenitude of Actinobacteria and Tenericutes, and reduce serum TMAO levels in rats with cardiac dysfunction (103). The prospect of traditional Chinese medicine targeting TMAO in the treatment of AS is promising; however, randomized controlled trials are still needed.

## Prospect

**Figure 1:**
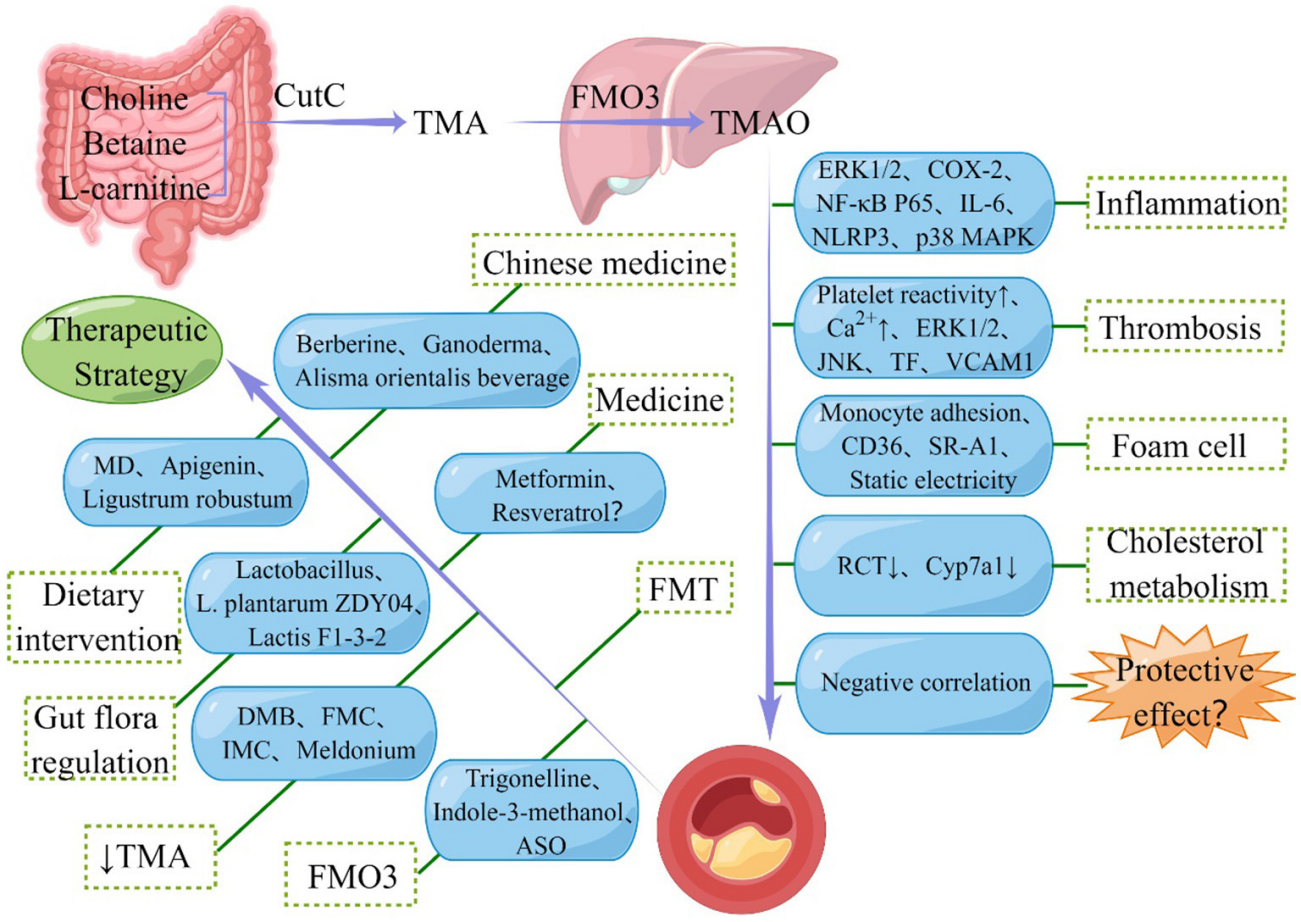
Schematic representation of the relationship between Trimethylamine N-oxide (TMAO) and atherosclerosis (AS). TMAO is synthesized by oxidation of trimethylamine (TMA) produced by gut microbes and promotes the development of AS through various pathways. However, some studies also suggested the protective effect of TMAO in AS. In addition, seven types of therapeutic strategies for alleviating AS targeting TMAO are also shown in the figure. CutC, choline TMA lyase; FMO3, flavin-containing monooxygenase-3; ERK1/2, extracellular signal-regulated kinase 1 and 2; COX-2, cyclooxygenase 2; NF-κB, nuclear factor kappa-B; IL-6, interleukin 6; NLRP3, nucleotide-binding oligomerization domain-like receptor family pyrin domain-containing 3; MAPK, mitogen-activated protein kinase; JNK, Jun N-terminal kinase; TF, tissue factor; VCAM, vascular cell adhesion molecule; SR-A1, scavenger receptor class A1; RCT, reverse cholesterol transport; Cyp7a1, cholesterol 7α-hydroxylase; FMT, fecal microbiota transplantation; MD, Mediterranean diet; DMB, 3, 3-Dimethyl-1-butanol; FMC, fluoromethylcholine; IMC, iodomethylcholine; ASO, antisense oligonucleotide.

TMAO can stabilize cellular proteins in marine organisms (104) and protect proteins against hydrostatic and osmotic stresses. Based on the relationship between CVD and hydrostatic pressure and osmotic pressure, some researchers have speculated that the increase in TMAO in CVD is a compensatory response. More and more evidence shows that TMAO, a metabolite of the intestinal flora, is closely associated with AS. The Human Microbiome Project, the development of high-throughput technologies, including bioinformatics and metabolomics, recently provided a deeper understanding of the important role of changes in the composition of the gut microbiota and its metabolites in causing AS in humans (105, 106). TMAO can promote the occurrence and development of AS by activating the inflammatory cascade, increasing the risk of thrombosis, promoting the formation of foam cells, and reducing the metabolism of cholesterol (Figure 1). Therefore, TMAO could be used as a novel target for the prevention and treatment of AS.

However, before that happens, specific issues related to current research must be sorted. Of the pathological mechanisms presented above, whether TMAO directly interacts with specific receptors or indirectly alters the signal pathway by changing the protein conformation remains unclear. More in-depth investigations into the mechanism of how TMAO affects AS must still be conducted. Current publications have shown that interventions, such as dietary management, intestinal flora regulation, the inhibition of TMAO precursor production, the prevention of TMA transformation, fecal flora transplantation, and treatment with medicine or traditional Chinese medicine, can prevent and treat AS. However, the signal transduction pathway of TMAO when promoting AS is not abundantly clear, and the types of bacteria currently identified are also relatively limited. To truly realize the prevention and treatment of AS with TMAO as the target, multiple multi-centers, large-scale clinical trials, as well as in-depth basic experimental evaluations, must be performed.

## Author Contributions

All authors contributed to the article and approved the submitted version.

## Funding

This research was funded by Jiaxing Public Welfare Projects (Grant no. 2022AD30058), the Key Medicine Disciplines Co-construction Project of Jiaxing Municipal (Grant no. 2019-ss-xxgbx), Pioneer Innovation Team of Jiaxing Institute of Atherosclerotic Diseases (Grant no. XFCX–DMYH), Program of the First Hospital of Jiaxing (Grant no. 2021-YA-011), and Jiaxing Key Laboratory of Arteriosclerotic Diseases (Grant no. 2020-dmzdsys).

## Conflict of Interest

The authors declare that the research was conducted in the absence of any commercial or financial relationships that could be construed as a potential conflict of interest.

## Publisher's Note

All claims expressed in this article are solely those of the authors and do not necessarily represent those of their affiliated organizations, or those of the publisher, the editors and the reviewers. Any product that may be evaluated in this article, or claim that may be made by its manufacturer, is not guaranteed or endorsed by the publisher.

## Abbreviations

CVD: cardiovascular disease
TMAO: Trimethylamine N-oxide
AS: atherosclerosis
FMO3: flavin-containing monooxygenase-3
DMB, 3: 3-Dimethyl-1-butanol.
